# Identification of 4-Trimethylaminobutyraldehyde Dehydrogenase (TMABA-DH) as a Candidate Serum Autoantibody Target for Kawasaki Disease

**DOI:** 10.1371/journal.pone.0128189

**Published:** 2015-05-26

**Authors:** Atsuko Matsunaga, Yutaka Harita, Yoshio Shibagaki, Nobutaka Shimizu, Kazuhiko Shibuya, Hiroshi Ono, Hitoshi Kato, Takashi Sekine, Naoko Sakamoto, Takashi Igarashi, Seisuke Hattori

**Affiliations:** 1 Division of Cellular Proteomics, Institute of Medical Science, The University of Tokyo, Minato-ku, Tokyo, Japan; 2 Division of Biochemistry, School of Pharmaceutical Sciences, Kitasato University, Minato-ku, Tokyo, Japan; 3 Department of Pediatrics, Graduate School of Medicine, The University of Tokyo, Bunkyo-ku, Tokyo, Japan; 4 Tokyo Metropolitan Children’s Medical Center, Fuchu, Tokyo, Japan; 5 National Center for Child Health and Development, Setagaya-ku, Tokyo, Japan; 6 Department of Pediatrics, Ohashi Hospital, Toho University School of Medicine, Meguro-ku, Tokyo, Japan; LMU Munich, GERMANY

## Abstract

Kawasaki disease (KD), an acute vasculitis that preferentially affects coronary arteries, is still the leading cause of acquired heart disease in children. Although the involvement of immune system malfunction in the onset of KD is suggested, its etiology still remains to be clarified. We investigated autoantibodies in KD patients, which are frequently found in sera from patients with autoimmune diseases, vasculitides and arteritides. We performed two-dimensional western blotting and LC-MS/MS to analyze the antigens of autoantibodies, detected two protein spots with 4 out of 24 sera from KD patients but not with 6 control sera, and identified the antigens as 4-trimethylaminobutyraldehyde dehydrogenase (TMABA-DH). A slot blot analysis with TMABA-DH as an antigen also revealed higher reactivities of patients' sera than control sera (positive rates: 18/43 vs 3/41). Using an enzyme-linked immunosorbent assay (ELISA), we found that the reactivity of anti-TMABA-DH antibodies in sera from KD patients was significantly higher than that in sera from age-matched controls. The optimal cut-off value of 0.043 had a sensitivity of 83.7% and a specificity of 80.0% in detecting KD patients (positive rates: 37/43 for KD patients, 9/41 for controls). Immunohistochemistry performed on thin sections of rat heart revealed that TMABA-DH colocalized with myosin light chains in cardiac myocytes. Patient sera with high reactivity gave similar immunostaining pattern. These results suggest that the detection of anti-TMABA-DH autoantibody could be a potential strategy for a diagnosis of KD.

## Introduction

Kawasaki disease (KD) is an acute systemic inflammatory vasculitis in children [1]. The diagnostic criteria for KD are fever for five days or more, bilateral, non-exudative conjunctivitis, erythema of the lips and oral mucosa, changes in the extremities, rash and cervical lymphadenopathy [2]. While its symptoms mimic many other childhood febrile illnesses, KD remains a major medical problem because coronary artery lesions such as aneurysms or ectasia develop in 20–25% of untreated children, which often leads to myocardial infarction, sudden death or ischemic heart disease [3]. Treatment with high-dose intravenous immunoglobulin reduces the risk of coronary artery lesions, however, 10–15% of KD patients are not responsive to intravenous immunoglobulin treatment [4]. KD is still the major cause of acquired heart disease in children in developed countries [5].

Although the etiology of KD remains to be clarified, clinical features of KD are suggestive of infectious agents. The aforementioned diagnostic criteria, the self-limited disease course, age and seasonal occurrence of KD are consistent with an infectious disease [5]. Superantigens produced by certain bacteria have also been implicated in the pathogenesis of KD [6]. Expansion of T cells with specific subsets of T cell receptors in KD patients suggests that some superantigen(s) are triggering the response [7]. Despite many studies, however, no known pathogen has been consistently identified in KD patients.

Innate and acquired immune responses may also be involved in the pathogenesis of the disease. Cytokines produced in the acute febrile phase are reminiscent of the innate immune response [8]. The oligoclonal expansion of T and B lymphocytes is also indicative of an acquired immune response [6, 7, 9]. Infiltration of IgA-producing plasma cells into vascular tissue suggests that these cells are responding to a specific antigen [10–12].

The high incidence rate in East Asian countries and high risk among siblings suggest that genetic factors also contribute to the onset of KD. Sibling pair studies identified a single nucleotide polymorphism (SNP) in the *CD40 ligand* (*CD40L*) gene that shows an association with coronary artery lesions in male KD patients [13]. SNPs in *ITPKC* (*inositol1*,*4*,*5-triphosphate (IP3) kinase C*) and *CASP3* genes that confer susceptibility to KD have also been identified [14, 15]. A genome-wide association study identified three novel risk loci for KD in the intragenic region between *FAM167A* and *BLK*, the *human leukocyte antigen* (*HLA*) region and the *CD40* region [16]. Interestingly, all risk alleles described above cause hyperactivation of T and B cell signaling pathways, suggesting that a deregulated immune response is one of the causes of KD.

Taken together, these findings suggest that KD may be brought on when many different events trigger the initial immune activation, which may result in the common pathological hyperimmune response observed in certain populations of genetically susceptible children.

An abnormal immune response may cause the production of autoantibodies, which are widely detected in various autoimmune/inflammatory diseases. Anti-neutrophil cytoplasmic antibodies (ANCA) [17–19] and anti-endothelial cell antibodies (AECA) [20–22] have been described in sera of patients with systemic vasculitis and vasculitis-associated diseases. It has been reported that AECA are also present in sera of KD patients [23–25]. Indeed, AECA in KD patient sera showed cytotoxicity against human umbilical vein endothelial cells (HUVEC) pretreated with IL-1, TNF-α or interferon-γ [23, 24], raising the possibility that these autoantibodies are involved in the development of vasculitis. These ANCA and AECA have been shown to be useful in diagnosis and prognosis of various autoimmune/inflammatory diseases. Major antigens for ANCA are myeloperoxidase [18] and proteinase 3 [19], however, antigens of AECA have not been fully elucidated. Proteomic techniques are useful to identify antigens of disease-specific autoantibodies, and have been applied to identify antigens for AECA in a few diseases including KD [26–28].

Previously, we carried out two-dimensional (2-D) western blot analyses to globally identify disease-specific autoantibodies and their antigens. Using this strategy, we analyzed autoantibodies in sera of patients with systemic lupus erythematosus, and identified 11 antigens that are the targets of autoantibodies [29, 30]. Interestingly, the level of one of the autoantibodies correlates with psychiatric syndromes [30]. We also demonstrated that IgA autoantibody against a mitochondrial protein, dihydrolipoamide dehydrogenase, is induced in patients with endometrial cancer [31].

In this study, we performed 2-D western blotting using HUVEC lysates as antigens and sera from KD patients as primary antibodies, and identified 4-trimethylaminobutyraldehyde dehydrogenase (TMABA-DH) as an antigen for autoantibodies detected in KD patients. We demonstrated that the reactivity of anti-TMABA-DH was significantly higher in KD patients using an ELISA, suggesting that anti-TMABA-DH autoantibody could be a novel diagnostic marker for KD. Interestingly, immunohistochemistry performed on thin sections of rat heart revealed that TMABA-DH colocalized with myosin light chains in cardiac myocytes.

## Materials and Methods

### Patients and sera

The Ethics Committee of Graduate School of Medicine and Faculty of Medicine, The University of Tokyo approved this project. All patients and their families gave their written consent to participate in this study. The Ethics Committee approved this consent procedure. The documents of consents were stored in a rocked room, which was managed by one of the authors (H. K.). Sera were obtained from 43 KD patients during the acute stage of the disease (before treatment). Patients with KD were hospitalized at the Omiya Medical Association General Hospital, the Tokyo Metropolitan Hachioji Children’s Hospital or The University of Tokyo Hospital. The 41 age-matched control sera were obtained at The University of Tokyo Hospital from congenital heart disease patients who were not feverish at the time. Detailed clinical data were also obtained from patients with KD using a retrospective chart review. Information on patient demographics, medical history, results of physical examinations, laboratory data, treatments and clinical course was collected.

### Cell culture

HUVEC were purchased from Lonza Walkersville (Walkersville, MD, USA). Cells were cultured on collagen I-coated culture dishes in EGM-2 medium supplemented with EGM-2 SingleQuots (TAKARA BIO, Shiga, Japan) and used between the second and ninth passages. Human embryonic kidney 293T (HEK293T) cells were cultured in DMEM medium supplemented with 10% (v/v) fetal bovine serum.

### 2-D gel electrophoresis and western blotting (2-D western blotting)

For 2-D western blotting with patient sera, 100 μg of solubilized proteins from HUVEC in a lysis buffer (7 M urea, 2 M thiourea, 4% CHAPS and 60 mM dithiothreitol (DTT)) were applied to an IPG strip (Immobiline, 13 or 24 cm Drystrips, 3–10 NL or 4–7 NL, GE Healthcare, Waukesha, WI, USA) and rehydrated for 12 h. Isoelectric focusing was performed under the following conditions; 500 V-500 Vhr, gradient 1,000 V-800 Vhr, gradient 8,000 V-11300 Vhr and 8,000 V-7400 Vhr. After isoelectric focusing, protein reduction was performed on the strip soaked in a buffer containing 50 mM Tris-HCl (pH8.5), 6 M urea, 30% (v/v) glycerol, 2% (w/v) SDS and 10 mg/mL DTT for 15 min. Alkylation was then performed in a fresh buffer containing 25 mg/mL iodoacetamide instead of DTT for 15 min. Protein separation in the second dimension was carried out using 9% SDS-PAGE. The resolved proteins were transferred electrophoretically onto a polyvinylidene difluoride (PVDF) membrane (Immobilon-FL) (Millipore, Billerica, MA, USA).

PVDF membranes were stained using Cy5 mono-reactive dye (GE Healthcare, 0.4 mg/mL in phosphate-buffered saline (PBS) containing 0.1% Tween 20 (PBS-T)) for 30 min to detect total proteins. After washing three times with methanol, the membranes were blocked with 5% skim milk in PBS-T overnight. Then the membranes were incubated for 3 h with individual control or KD patient sera at a dilution of 1:500 in PBS-T. The membranes were washed with PBS-T three times and incubated with peroxidase-conjugated anti-human IgG/IgA/IgM (Zymed Laboratories, South San Francisco, CA, USA) at a dilution of 1:10,000 for 1 h. The reacted spots were visualized with ECL plus (GE Healthcare), and the signals were scanned using a Typhoon 9400 imager (GE Healthcare) under the following conditions: excitation 633 nm/detection 670 nm for Cy5 and excitation 457 nm/detection 520 nm for ECL plus signals. Each serum was analyzed 3 to 5 times.

### In-gel digestion and protein identification

To identify proteins in the reacted spots, 300 μg of HUVEC proteins were separated by 2-D electrophoresis as described in the previous section. Then, the gels were fixed with 7.5% (v/v) acetic acid/10% (v/v) methanol overnight, stained with Deep Purple Total Protein Stain (GE Healthcare) for 1 h under dark and developed in a solution containing 0.1% ammonia. The gels were scanned on a Typhoon 9400 imager. After scanning gels, a spot-picking list was generated using Decyder software that was exported to an Ettan Spot Picker (GE Healthcare). Spots were excised as plugs and collected into 96-well microtiter plates. Gel pieces were washed twice each with water, 50% (v/v) acetonitrile and 100% (v/v) acetonitrile, digested overnight with 1 pmol lysylendopeptidase (Wako Pure Chemical Industries, Osaka, Japan) in 25 μL of 10 mM Tris-HCl (pH8.5) at 37°C. Peptides were extracted with 50 μL of 50% (v/v) acetonitrile/0.1% (v/v) TFA twice and 50 μL of 80% (v/v) acetonitrile 0.1% (v/v) TFA once. Then, extracts were combined and desalted using a C_18_ tip column (3M Empore, St. Paul, MN, USA).

LC-MS/MS analyses were performed using a nano-LC-MS/MS system (nano-LC, DiNa Nano-LC, KYA technologies, Tokyo, Japan; QSTAR Elite mass spectrometer, Applied Biosystems (Carlsbad, CA, USA)). The MS/MS data were searched against the UniprotKB human protein sequence database using ProteinPilot software version 2.0 (Applied Biosystems) with default parameters.

### Expression and Purification of TMABA-DH

Total RNA was isolated from HUVEC using ISOGEN (Nippon Gene, Toyama, Japan) and reverse transcribed into cDNA using SuperScript First-Strand Synthesis System for RT-PCR (Invitrogen, Carlsbad, CA, USA). The TMABA-DH cDNA was amplified using polymerase chain reaction (PCR), with the following oligonucleotides: forward, tatgaattcgccaccatgagcactggcaccttcg; reverse, tatgtcgacaaaagcagattccacatcaccc (the underlined sequences were artificially added sequences). The amplified TMABA-DH cDNA and pCMV-Tag4 vector (Stratagene, La Jolla, CA, USA) were digested with EcoRI and SalI and ligated. The resultant FLAG-TMABA-DH vector was transfected into HEK293T cells using Lipofectamine 2000 according to the manufacturer’s protocol. One day after transfection, cells were lysed with a lysis buffer (20 mM Tris-HCl, 150 mM NaCl, 1% NP40, 1% aprotinin, 0.1% leupeptin, 0.1% antipain, 50 mM NaF, 1 mM phenylmethylsulfonyl fluoride and 1 mM EDTA). FLAG-tagged TMABA-DH was immunopurified using Anti-FLAG M2 agarose (Sigma-Aldrich, St. Loius, Mo, USA) and was eluted from the immunoprecipitates twice with PBS containing 1 mM FLAG-peptide (Sigma-Aldrich, 100 μL/10 cm culture dish). RT-PCR was also carried out to quantify TMABA-DH in HUVEC or various tissues.

### ELISA

One hundred ng of FLAG-TMABA-DH in 100 μL of PBS was fixed on the bottom of each well of a 96-well microtiter plate by incubation overnight at 4°C. After blocking with a solution containing 5% bovine serum albumin (BSA) in PBS for 1 h, 100 μL of 1:2,000 diluted control or KD patient sera in the blocking solution was added to each well and incubated for 2 h at room temperature. After washing the wells with 100 μL of PBS three times, horseradish peroxidase-conjugated goat anti-human IgG/IgM/IgA at a dilution of 1:10,000 in the blocking solution was added to each well and incubated for 1 h. 3,3´, 5,5´-tetramethylbenzidine (SeeBlue Reserve TMB Microwell Peroxidase Substrate, KPL, Gaithersburg, MD, USA) was used for signal detection and the absorbance was determined at 450 nm using a microplate reader. The antibody reactivity of each serum was measured using 5 wells of the ELISA plates simultaneously, and the mean value was employed.

Anti-alpha-smooth muscle actin (α-SMA) was detected using a commercially available kit (human anti SMA ELISA Kit (CSB-E09419h, CUSABIO, Wuhan, Hubei, R.P. China)) according to the manufacturer’s protocol. One hundred μL of 1:11 diluted control or KD patient sera in the sample diluent were added to each well. Three wells were used for each serum and the mean value was employed.

### Statistical analysis

Two-sample *t*-test assuming unequal variances was performed by EXCEL 2011. *P* values less than 0.05 were considered to be statistically significant. Receiver operating characteristic (ROC) curve was drawn and area under the curve (AUC) was calculated by EXCEL software equipped with XLSTAT (Addinsoft, New York, USA).

### Immunohistochemistry

HUVEC cultured on collagen I-coated glass coverslips (12 mm, Iwaki Glass Co., Ltd, Chiba, Japan) were treated with 0.37% formaldehyde in PBS for 10 min, ice-cold 10% trichloroacetic acid for 15 min, and 0.1% Triton X-100 in PBS for 5 min. After blocking with 2% BSA in PBS, cells were incubated with anti-TMABA-DH antibody (rabbit polyclonal, #HPA010873, anti-ALDH9A1, Sigma-Aldrich, 1/65 dilution) for 60 min at 37°C. The cells were washed and incubated with FITC-conjugated anti-rabbit IgGs (1/1,000) in the blocking solution for 1 h at 37°C, mounted with ProLong Gold Antifade reagent with DAPI (4',6'-diamidino-2-phenylindole, Invitrogen) and examined using a fluorescence microscope. The experiments were carried out for three times.

The animal experiments were approved by The Animal Research Committee of Graduate School of Medicine, The Tokyo University, and were carried out according to the guidelines of the Committee. Rat heart slices (5 μm thick) were prepared using a cryostat, fixed with acetone for 1 min and dried. Slices were blocked successively with 10% goat serum, 2% BSA and 0.4% Triton X-100 in PBS and incubated with an appropriate primary antibody in the blocking solution (anti-TMABA-DH, 1/10 dilution; anti-myosin light chain antibody (mouse monoclonal, IgM, anti-MLC, ab11082, Abcam, Cambridge, UK) 1/200; anti-α-SMA (mouse monoclonal, IgG_2a_, A2547, Sigma-Aldrich) 1/200; control or KD patient sera, 1/50) for 1 h at room temperature. After rinsing with PBS-T three times, the slices were incubated with an appropriate secondary antibody conjugated with Alexa Fluor 488 or 555 (Invitrogen, 1/500) for 1 h, and mounted with ProLong Gold Antifade reagent with DAPI. The experiments were performed 3 times using two rats.

## Results

### Identification of autoantibodies

**Fig 1:**
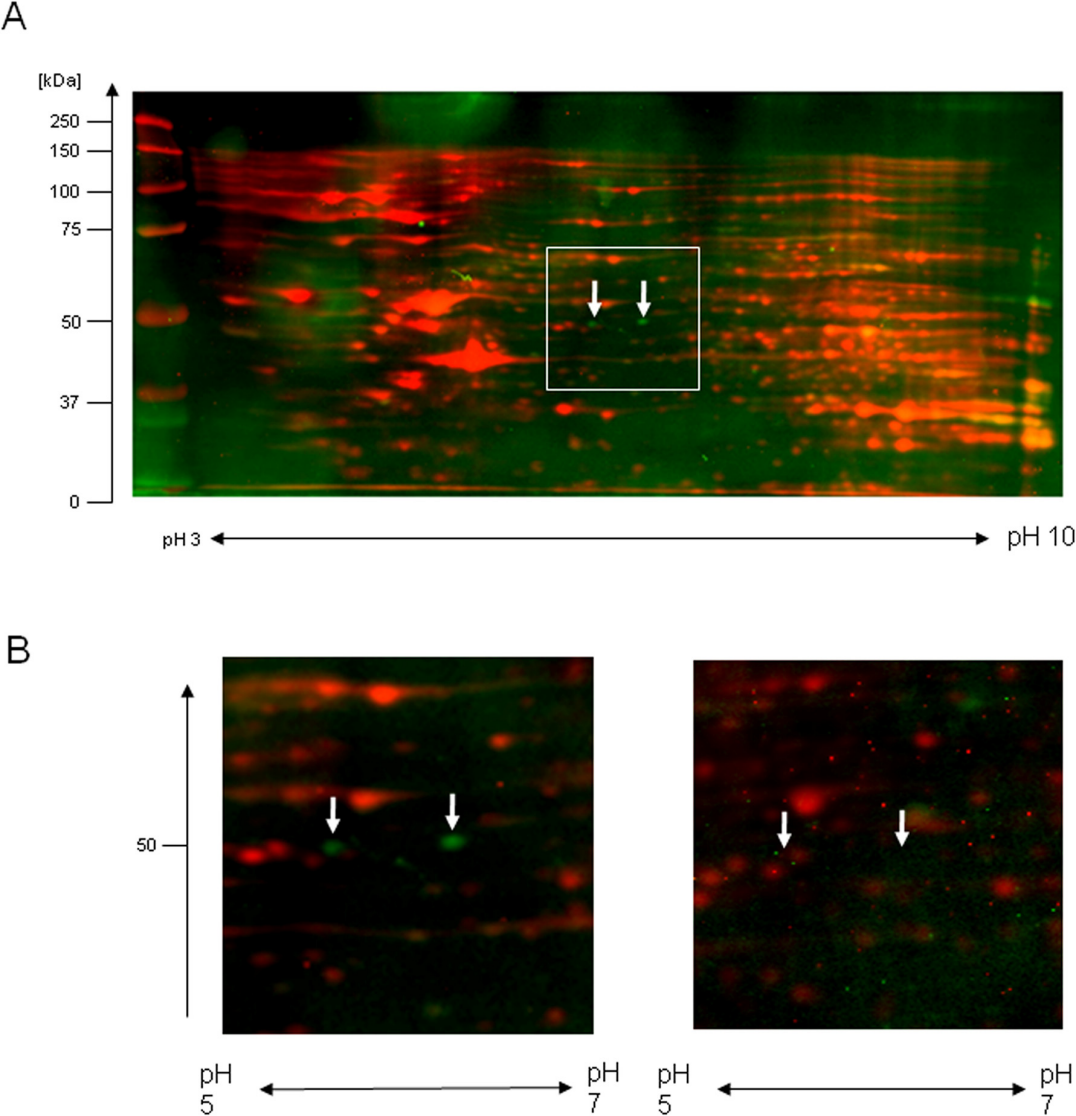
Detection of autoantigens with sera from KD patients. (A) 2-D western blot analyses were performed as described under Materials and Methods, and a typical result obtained using sera from a KD patient is shown. Western blot signals detected using ECL plus are shown by green pseudocolor and total protein profile detected using staining with Cy5 dye is shown by the red pseudocolor, and the two signals were merged. Arrows demonstrate spots that were detected with sera from multiple KD patients. Each serum was analyzed 3–5 times. (B) A magnified view of the area containing the detected spots (indicated by a white box in (A)) is shown for the same patient and for an antibody-negative patient.

To search for autoantibodies induced in patient sera, 2-D western blotting was performed with HUVEC lysates as antigens using each of twenty-four sera from KD patients or six sera from age-matched congenital heart disease patients. Multiple protein spots were detected on 2-D western blotting with sera from both KD patient and control sera, however, most of the spots were common between KD patients and controls. Two protein spots were detected with sera from four KD patients, but not with control sera, proteins that were considered to be autoantigens for autoantibodies induced in KD patients (Fig 1A and 1B). Their molecular weight and isoelectric point were approximately 50 kDa and 6.0, respectively.

### Identification of autoantigens

**Fig 2:**
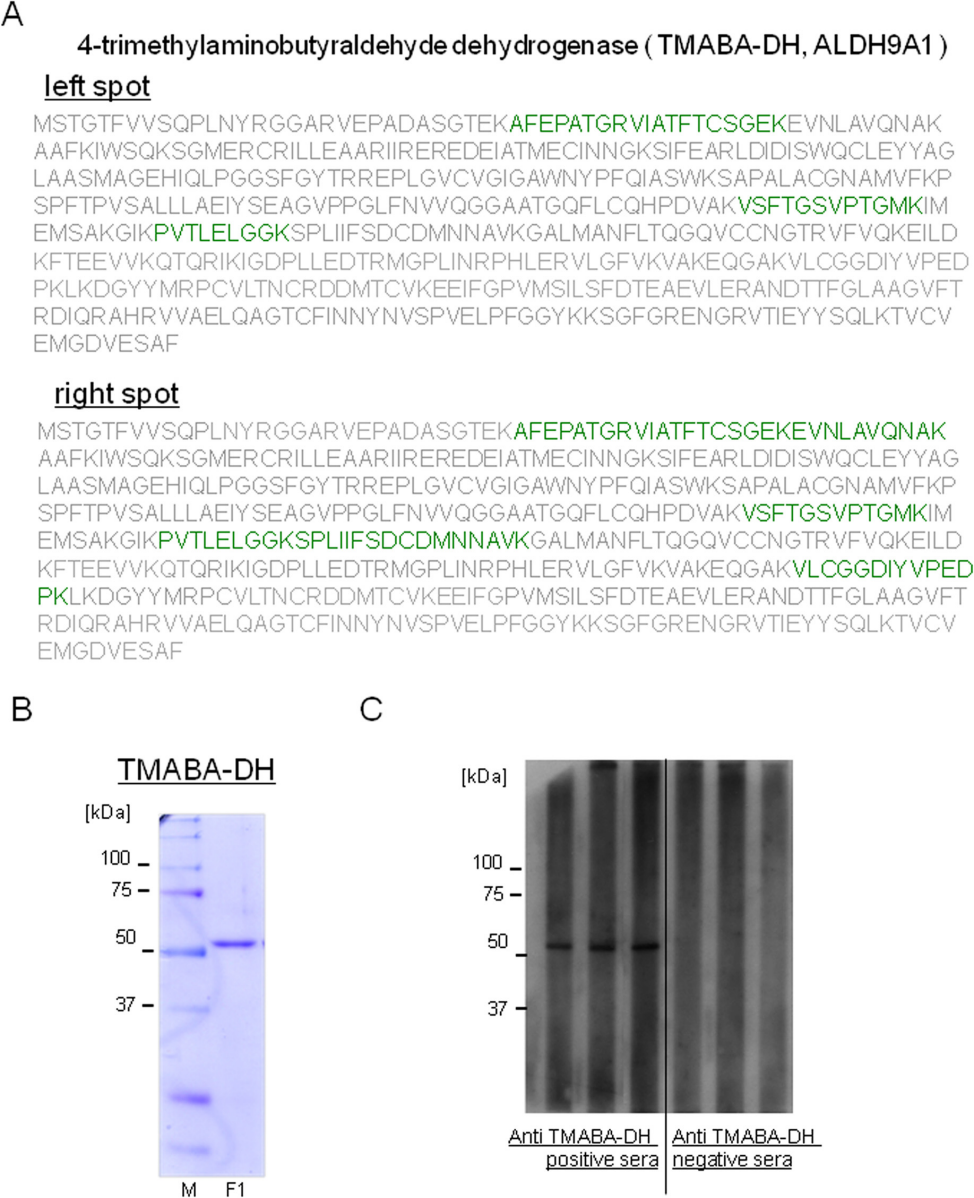
Identification of the autoantigen as TMABA-DH. (A) Proteins in the spots indicated by arrows in Fig 1 were excised and subjected to lysylendopeptidase digestion. The resultant peptides were analyzed by LC-MS/MS as described under Materials and Methods. Proteins in both spots were identified as TMABA-DH (peptides identified using LC-MS/MS with >99% confidence are shown by green characters). (B) FLAG-tagged TMABA-DH was expressed in HEK293T cells, immunopurified using anti-FLAG M2 beads and competitively eluted using a solution containing FLAG peptide. An aliquot of the purified sample was analyzed using 9% SDS-PAGE followed by staining with coomassie brilliant blue. Molecular weight markers and their size in kilodaltons are shown on the left side of the figure. (C) Results of slot blot analyses of anti-TMABA-DH positive and negative sera are shown. Slot blot analyses were performed 5 times and the representative results are shown.

To identify the proteins in the reactive spots, HUVEC proteins (300 μg) were subjected to 2-D electrophoresis and the gel was stained with Deep Purple Total Protein Stain. The two spots corresponding to those detected in 2-D western blotting were excised from the gel and proteins in the spots were digested with lysylendopeptidase. The resultant peptides were analyzed using an LC-MS/MS system, and the data were searched against the UniprotKB human protein sequence database. Four to six peptides were identified with 99% confidence from both spots (illustrated by green characters in Fig 2A), which enabled us to identify the proteins in both spots as 4-trimethylaminobutyraldehyde dehydrogenase (TMABA-DH) (Fig 2A) (the left spot; Protscore 6.47, four peptides covering 14.5% sequence: the right spot; Protscore 12.19, six peptides covering 26.5% sequence), also known as aldehyde dehydrogenase 9 family, member A1 (ALDH9A1). TMABA-DH is a cytosolic enzyme that catalyzes the irreversible oxidation of a broad range of aldehydes to their corresponding acids in an NAD-dependent reaction [31, 32].

### Evaluation of autoantibodies against TMABA-DH

We investigated the possibility that the autoantibody against TMABA-DH could be used as a diagnostic marker for KD. We constructed an expression vector for FLAG-tagged TMABA-DH (FLAG-TMABA-DH) and transfected the plasmid into HEK293T cells. FLAG-TMABA-DH was immunoprecipitated with anti-FLAG M2 agarose beads and competitively eluted from the beads with 1 mM FLAG peptide. The analysis of the eluates with SDS-PAGE demonstrated that the protein was purified to near homogeneity (Fig 2B).

**Fig 3:**
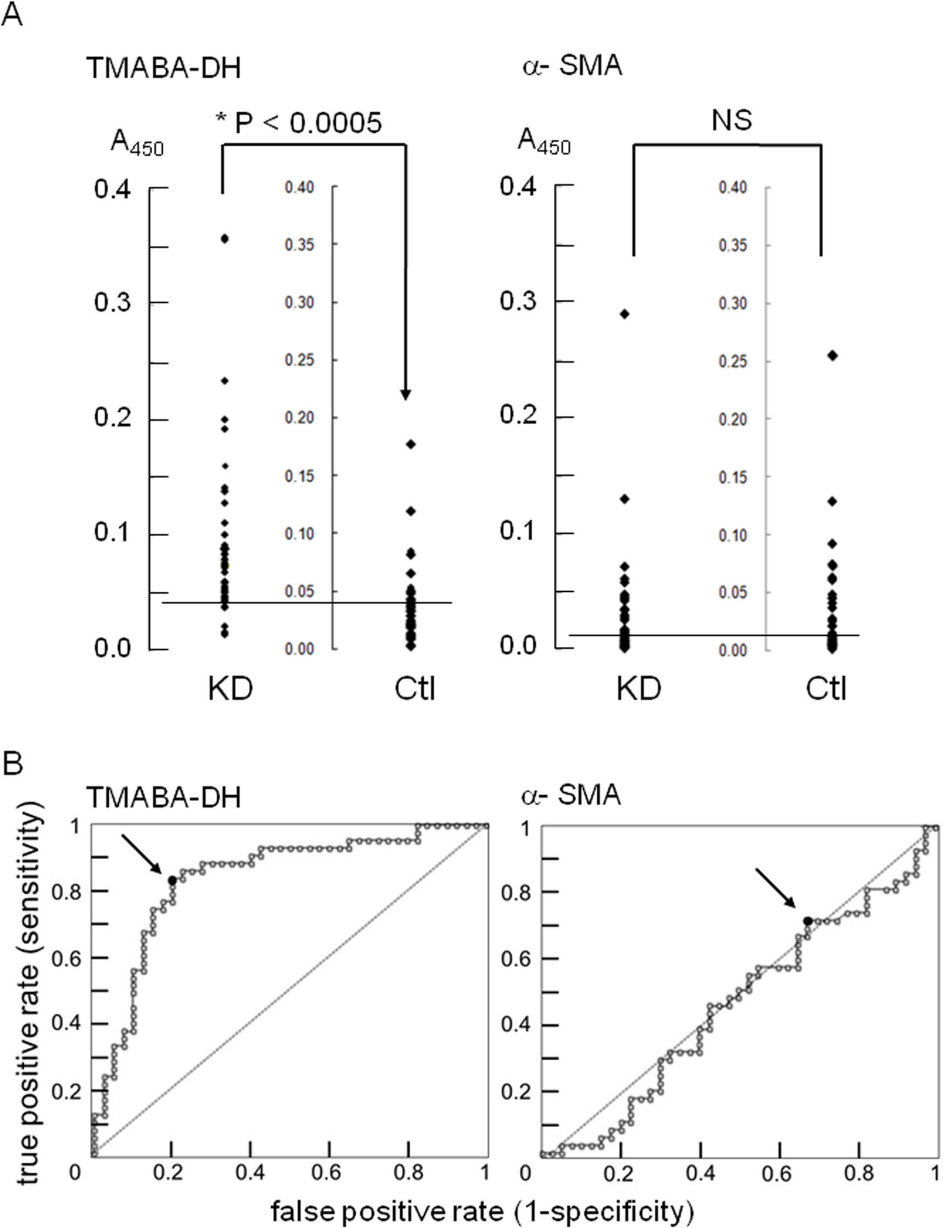
Quantitation of antibodies against TMABA-DH and α-SMA in sera from patients with KD or controls. (A) Data are presented as scattergrams for KD patients (KD) and for controls (Ctl). The difference between the reactivity of the anti-TMABA-DH antibody in KD patients and control patients is statistically significant (P<0.0005), while the reactivity of the anti-α-SMA antibody did not differ between the two groups (NS, not significant; *P*>0.45). Horizontal lines in the figure represent cut-off values for the anti-TMABADH antibody and the anti-α-SMA antibody, 0.043 and 0.007, respectively. Typical data from 5 independent measurements are shown. (B) ROC curve analyses of anti-TMABA-DH and anti—SMA antibody reactivity in (A) with AUC values of 0.84 and 0.47, respectively. Closed circles (designated by arrows) show the optimal cut-off points.

We first carried out a slot blot analysis using purified FLAG-TMABA-DH as an antigen to evaluate the autoantibodies in sera from KD patients (Fig 2C). In this analysis, 18 of 43 sera from KD patients (41.9%) reacted with the antigen, whereas only 3 of 41 sera from age-matched control sera (7.3%) gave positive signals. To measure the reactivity of the autoantibodies in KD patients more precisely, we next performed an ELISA and receiver operating characteristic (ROC) analysis using the same antigen (Fig 3A). The reactivity of the anti-TMABA-DH antibody in KD patient sera was significantly higher than that in sera from age-matched controls (*P*<0.0005).

As a control experiment we carried out an ELISA using anti-alpha smooth muscle actin (α-SMA) as the antigen. The reactivity of anti-α-SMA did not show any difference between KD patients and age-matched controls (*P*>0.45). Raw data of Fig 3A are available in S1 Table. We also measured antibody reactivities to ovalbumin as another control experiment. Again, the reactivity of anti-ovalbumin was not different between KD patients and age-matched controls (*P*>0.3) (data not shown).

We then performed receiver operating characteristic (ROC) analysis to verify the usefulness of the anti-TMABA-DH antibody as a diagnostic marker. The ROC curve of anti-TMABA-DH antibody was much closer to the upper left corner than that of anti-α-SMA antibody, and the area under the curve (AUC) of anti-TMABA-DH and anti-α-SMA antibodies was 0.84 and 0.47, respectively (Fig 3B). The optimal cut-off value of 0.043 had a sensitivity of 83.7% and a specificity of 80.0% in detecting KD patients (positive rates: 37/43 for KD patients, 9/41 for controls), whereas the optimal cut-off value for anti-α-SMA antibody was 0.007 with a sensitivity of 72.1% and a specificity of 32.5% (12 of 43 sera from KD patients, 12 of 41 sera from age-matched controls), suggesting anti-TMABADA antibody could be a candidate marker for KD diagnosis.

### Expression and localization of TMABA-DH

Autoantibodies are considered to be one of the causes in various kinds of diseases [17–26]. As we found that the anti-TMABA-DH antibody was induced in KD patients, we examined whether this autoantibody was involved in the development of the disease. To address this issue, it was necessary to investigate tissue distribution and cell type specificity of TMABA-DH.

**Fig 4:**
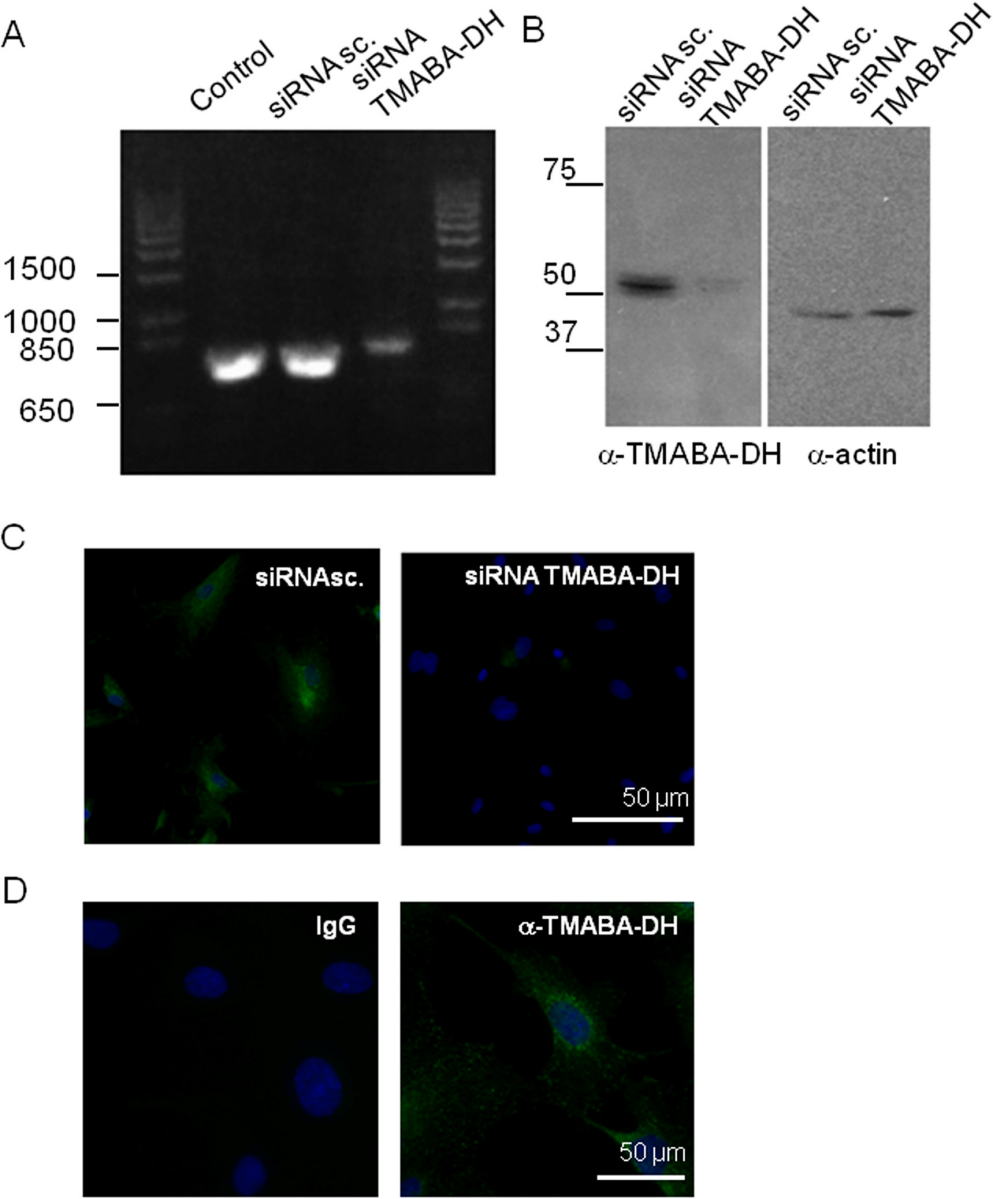
Specificity of an anti-TMABA-DH antibody. siRNA treatment of HUVEC significantly decreased the levels of TMABA-DH mRNA detected by RT-PCR and agarose gel electrophoresis (A) and protein in immunoblot analysis (B), and antibody reactive signals using immunohistochemistry (C), as compared with control or scrambled siRNA (siRNA sc.) treatment. In (C), nuclei were stained with DAPI (Blue). In (D), HUVEC were immunostained with the antibody (green) or control IgG with nuclei being stained with DAPI (blue). Bars indicate 50 μm. The experiments were repeated 3 times.

To investigate the expression profile of TMABA-DH *in vivo*, we first evaluated the specificity of a commercially available anti-TMABA-DH antibody (anti-ALDH9A1, Sigma-Aldrich) (Fig 4). HUVEC abundantly expressed the TMABA-DH mRNA and the encoded protein. When we decreased the level of TMABA-DH mRNA using siRNA (Fig 4A), TMABA-DH protein detected with the antibody on western blotting was significantly decreased (Fig 4B). siRNA treatment also abrogated the immunofluorescent staining of TMABA-DH in HUVEC (Fig 4C). Nonspecific IgG did not give any signals under the same conditions (Fig 4D). These results confirmed the specificity of this antibody.

### Expression of TMABA-DH in heart tissues

We also examined the expression of TMABA-DH in various rat tissues and several human cell lines by RT-PCR and western blotting. TMABA-DH was ubiquitously expressed in all tissues and cell lines examined, with high expression in kidney and liver, and moderate expression in other tissues (data not shown), consistent with the distribution of the enzymatic activity [32].

**Fig 5:**
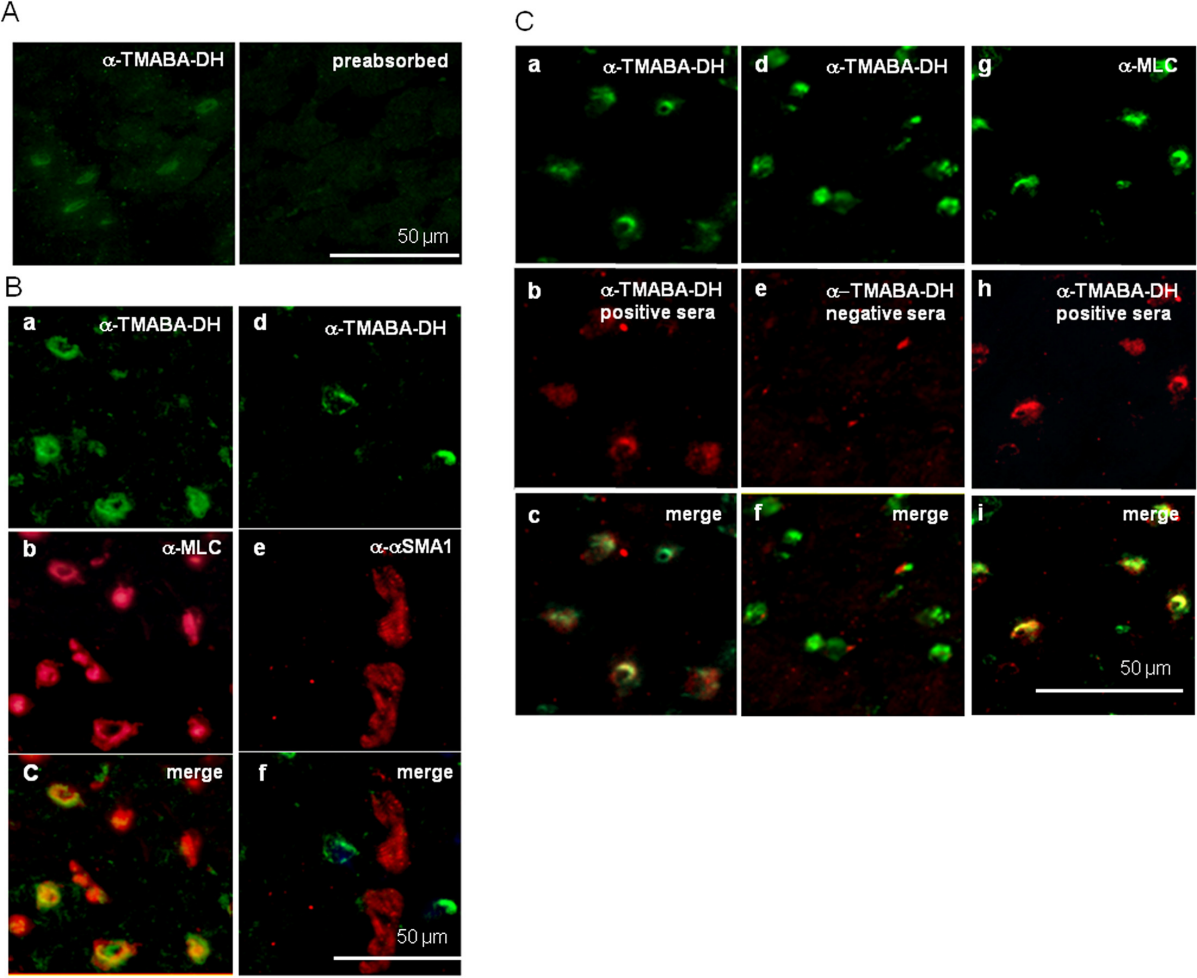
Immunohistochemistry of rat heart thin sections. (A) Immunostaining of rat heart tissue with the anti-TMABA-DH antibody (Sigma, green) (left) or the same antibody pre-absorbed with FLAG-TMABA-DH-bound M2 beads. (B) Co-staining with anti-TMABA-DH (green) and anti-myosin light chain (MLC, red) antibodies (a-c), and with anti-TMABA-DH (green) and anti-α-SMA (red) antibodies (d-f). In (C), co-staining with the anti-TMABA-DH (green) antibody and anti-TMABA-DH-positive sera (red) (a-c), the anti-TMABA-DH antibody (green) and anti-TMABA-DH-negative sera (red) (d-f), and anti-MLC antibody (green) and anti-TMABA-DH-positive sera (red) (g-i). Bars indicate 50 μm. Immunohistochemistry experiments were performed 3 times.

KD is characterized by acute febrile vasculitis and myocarditis that often results in coronary artery aneurysms with cardiac complications [33]. It has been suggested that autoimmunity may be involved in the pathogenesis of various heart diseases [34]. Indeed, patients with KD had significantly higher anti-myosin IgM autoantibody reactivities than controls [35]. Therefore, we immunostained rat heart thin sections with the anti-TMABA-DH antibody (anti-ALDH9A1, Sigma-Aldrich) and sera from patients that were positive for the anti-TMABA-DH autoantibodies to address a possible role of the autoantibody to TMABA-DH in the onset of KD (Fig 5). The anti-TMABA-DH antibody immunostained a population of the cells, and the reactivity was abrogated when the antibody was pre-absorbed with purified TMABA-DH from HEK293T cells (Fig 5A). When rat heart sections were co-stained with anti-TMABA-DH and anti-myosin light chain (a marker for cardiac myocytes) antibodies, most of the signals were found to be co-localized, suggesting that the cells positive for TMABA-DH signals were mainly cardiomyocytes (Fig 5B a-c).α-smooth muscle actin (α-SMA), a marker for endothelial cells, did not colocalize with TMABA-DH signals (Fig 5B d-f).

Next, we immunostained rat heart tissues with sera from KD patients that showed strong immunoreactivity to TMABA-DH (Fig 5C). The immunosignals detected by patient sera coincided well with those detected using the commercial antibody (Fig 5C a-c), whereas sera that lacked anti-TMABA-DH antibodies did not show such immunoreactivity (Fig 5C d-f). The myosin light chains also colocalized with signals detected using patient sera that were positive for the anti-TMABA-DH autoantibodies (Fig 5C g-i). These results confirmed that patient sera contained autoantibodies to TMABA-DH.

## Discussion

It has been shown that autoantibodies are induced in various systemic vasculitides [17–28]. In KD, AECA have been reported to show cytotoxic activity by binding to endothelial surface antigens [23–25], which is enhanced by the pretreatment of HUVEC with various inflammatory cytokines such as tumor necrosis factor, interleukin-1 and γ-interferon [23, 24]. Although the biological significance of these autoantibodies has been documented, autoantigens for the autoantibodies in KD patients have not been well characterized. Therefore, it is interesting to globally identify such autoantigens employing a recently developed proteomic approach.

In this study, we have shown that the anti-TMABA-DH antibody is significantly induced in sera of KD patients. The reactivity of anti-TMABA-DH antibodies quantitatively evaluated by an ELISA was much higher in sera from KD patients than in those from controls, and ROC curve for the anti-TMABA-DH antibody was much higher than that for the anti-α-SMA antibody, suggesting that this antibody may be the potential candidate as a novel marker for a diagnosis of KD. The positive rates observed in 2-D western blotting (4/24) and slot blot (18/43) analyses were lower than that in Elisa (37/43). Two possibilities could account for the difference in the positive rates. Whereas denatured antigen was employed in the former two assays, native protein was used in the Elisa. In addition, ELISA is more sensitive than western blot and western blot is more specific than ELISA in general. Although we have succeeded in the identification of anti-TMABA-DH autoantibody as a candidate marker for KD diagnosis, the sensitivity and specificity of the assay should be carefully evaluated and improved for more precise diagnosis of the disease. The use of europium-labeled nanoparticle as a tracer and time-resolved fluorometric immunoassay may improve sensitivity of the detection of antibody [36].

TMABA-DH (ALDH9A1) is a member of the aldehyde dehydrogenase family (ALDHs) that catalyzes the oxidation of a wide spectrum of endogenous and exogenous aliphatic and aromatic aldehydes [32, 37]. Thus far, 16 ALDH genes with distinct chromosomal locations have been identified in the human genome [38]. Polymorphisms in ALDH3A2, ALDH4A1, ALDH5A1 and ALDH6A1 are associated with metabolic diseases accompanying neurologic complications [38–40] and inactivating mutations in ALDH3A2, ALDH4A1, ALDH5A1 and ALDH6A1 cause Sjögren-Larsson syndrome, type II hyperprolinemia, 4-hydroxybutyric aciduria and developmental delay, respectively [38, 41, 42]. Allelic variants in ALDH9A1 gene have also been observed [38], which is recently shown to be weakly associated with neuroleptic-induced tardive dyskinesia. [43] Amino acid sequences of TMABA-DH identified in this study matched the canonical sequence.

It has been suggested that abnormal immune response may be involved in the pathogenesis of KD [3, 10–12]. As a manifestation of the abnormal immune response, various autoantibodies are induced in KD patients [23–25, 34]. To examine a possible involvement of autoantibody to TMABA-DH in the pathogenesis of KD, we carried out immunohistochemical analyses on rat heart thin sections using the commercial antibody against TMABA-DH (Fig 5). The signals detected by anti-TMABA-DH antibody colocalized with the myosin light chains but not with α-smooth muscle actin (α-SMA), suggesting that TMABA-DH was expressed mainly in cardiac myocytes rather than endothelial cells. When HUVEC were immunostained without Triton X-100 treatment, the anti-TMABA-DH antibody did not react with the antigen (data not shown), indicating that TMABA-DH is not exposed at the cell surface. However, proteins located inside of cells often translocate to the plasma membrane upon treatment with inflammatory cytokines [44, 45], which may mimic disease conditions. Reactivity of KD patient sera to endothelial cells was also augmented when the cells were treated with cytokines [23, 24]. Whether cardiac myocytes or endothelial/smooth muscle cells express TMABA-DH at the cell surface under disease conditions is a very intriguing issue to be investigated.

We also examined whether the reactivity of the anti-TMABA-DH antibody correlated with any laboratory measurements (age, white blood cell (WBC), neutrophil counts or platelet counts; or serum concentrations of aspartate aminotransferase (AST), alanine aminotransferase (ALT), sodium, albumin or C-reactive protein (CRP) levels). However, none of these data showed significant correlation except for sodium ion concentration that showed a weak negative correlation (P = -0.04). The sample size was not large enough to evaluate whether the antibody reactivity exhibited a relationship with responsiveness to intravenous immunoglobulin treatment.

We employed the age-matched control sera from congenital heart disease patients without fever. However, since KD is associated with fever and vasculitis, and it is considered that some pathological hyperimmune response is involved in the onset of KD, our age-matched control sera may not be the best controls. The use of sera from patients with different acute vasculitis, acute inflammatory conditions, and autoimmunity may serve as better controls in subsequent investigations.

Recently, proteomic methods have been applied to discover novel biomarkers for KD and other vasculitis diseases [28, 46–48]. Autoantibodies to peroxiredoxin 2 are induced in patients with KD [28]. Urine proteomics provides a noninvasive diagnosis for KD with high specificity and sensitivity [47]. These results, together with our findings described in this study, not only contribute to biomarker discovery, but also may also shed light on the mechanism of pathogenesis of these diseases.

## Supporting Information

S1 Table Raw data of ELISA shown in Fig 3A. Mean OD_450_ values measured in ELISA are shown.
(XLS)
